# Three-Dose Primary Series of Inactivated COVID-19 Vaccine for Persons Living with HIV, Hong Kong

**DOI:** 10.3201/eid2810.220691

**Published:** 2022-10

**Authors:** Denise Pui Chung Chan, Ngai Sze Wong, Bonnie C.K. Wong, Jacky M.C. Chan, Shui Shan Lee

**Affiliations:** The Chinese University of Hong Kong, Hong Kong, China (D.P.C. Chan, N.S. Wong, S.S. Lee);; Department of Health, Hong Kong (B.C.K. Wong);; Princess Margaret Hospital, Hong Kong (J.M.C. Chan)

**Keywords:** COVID-19, SARS-CoV-2, severe acute respiratory syndrome coronavirus 2, viruses, respiratory infections, zoonoses, inactivated vaccine, persons living with HIV, Hong Kong, HIV/AIDS, vaccines

## Abstract

In a cohort of persons living with HIV in Hong Kong, surrogate virus neutralization testing for COVID-19 yielded a median level of 89% after the third dose of an inactivated COVID-19 vaccine, compared with 37% after the second dose. These results support using a 3-dose primary series for enhanced immune protection.

Worldwide, inactivated vaccines are most widely used to prevent SARS-CoV-2 infection and severe COVID-19 disease (*1*). Vaccination effectiveness is of particular importance for protecting persons at increased risk for severe diseases, notably immunocompromised patients, including persons living with HIV (PLHIV). As recently reported in a prospective study in Brazil (*2*), immunogenicity of inactivated vaccine is lower in PLHIV than in healthy adults. This lower protection is a cause for concern, especially in populations with high burden of HIV/AIDS and COVID-19. In Hong Kong, both inactivated and mRNA vaccines are available free for all eligible healthy and immunocompromised citizens. Immunocompromised persons have been prioritized for receiving a third, booster, dose, 3 months after completion of a 2-dose series of any COVID-19 vaccine. In a real-world study conducted prospectively on PLHIV in Hong Kong, we measured vaccine immunogenicity by the surrogate virus neutralization test (sVNT) to compare the responses after completion of 2 versus 3 doses of CoronaVac (Sinovac, https://www.sinovac.com), the same inactivated vaccine used in the Brazil study (*2*). Based on antibody-mediated blockage of ACE2-spike receptor binding domain (RBD) interaction, the sVNT results were used to assess the amplitude of neutralizing antibody responses against SARS-CoV-2 (*3*,*4*).

During April 2021–March 2022, a total of 122 PLHIV who had received CoronaVac were enrolled at 2 major HIV specialist clinics providing comprehensive HIV care, including antiretroviral therapy, in Hong Kong. Participants provided informed consent. We measured sVNT after completion of 2 or 3 doses of CoronaVac, in addition to transcribing demographic and clinical data collected during routine clinical follow-up appointments (Appendix). The median age of recruited PLHIV was 49 (IQR 40–56.5) years of age; most (86%) were male, all were receiving antiretroviral therapy, and the median latest CD4 count was 564.5/μL (IQR 394–733/μL) (Appendix Table 1). We included in the analyses a total of 132 sVNT measurements made within 90 days (median 48 days, IQR 24–70 days) of the second and within 90 days (median 33 days, IQR 28–53 days) of the third dose. We expressed results as percentage inhibition, using a cutoff of 30% for positive neutralizing response. 

The median sVNT level was 37% (IQR 24%–53%); 64% of participants tested positive (sVNT >30%) after the second dose. After the third dose, the median sVNT rose to 89% (IQR 58%–95%; Mann-Whitney U = 648.5; p<0.001), paralleling a significantly higher percentage with sVNT positivity (91%; OR 5.67, 95% CI 1.86–17.33) (Figure). In multivariable linear regression, third-dose vaccination (B = 33.61; p<0.001), days past respective dose (B = −0.17; p = 0.047), and latest CD4 count (B = 0.02; p = 0.02) were significant factors associated with high sVNT, whereas viral load suppression (<200/mL) and age were not significant (Appendix Table 2).

**Figure:**
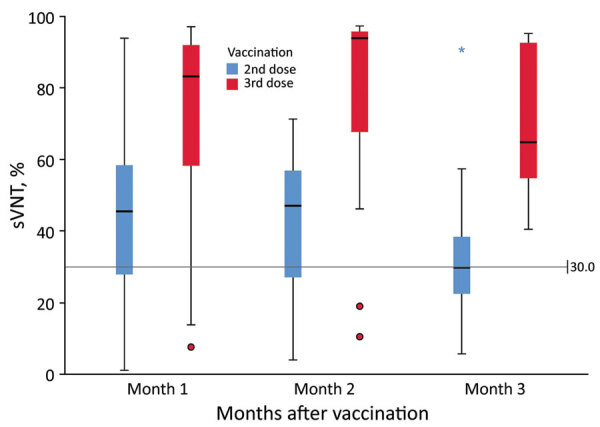
Distribution of sVNT by month after second and third doses of CoronaVac vaccine (Sinovac, https://www.sinovac.com) among persons living with HIV, Hong Kong. Horizontal lines inside boxes indicate medians, box tops and bottoms indicate 25th and 75th percentiles, and error bars indicate high and low values excluding outliers. Blue asterisk and red dots indicate outliers. Gray line indicates cutoff of 30% for positive neutralizing response. sVNT, surrogate virus neutralization test.

Our immunogenicity results on the completion of a 2-dose schedule of CoronaVac in PLHIV were remarkably similar to those reported in Brazil (*2*). After 2 doses of CoronaVac, 28 PLHIV in Hong Kong had a median sVNT of 48% (IQR 30%–58%) after 27–55 days, compared with median sVNT of 46.2% (IQR 26.9%–69.7%) in Brazil after 41 days. The corresponding proportion of PLHIV with sVNT positivity (>30%) was 79% after 27–55 days in our study and 71% after 41 days in the Brazil study. 

Although effectiveness of inactivated COVID-19 vaccines has previously been shown in PLHIV (*5*), their moderate efficacy and waning immunogenicity after a standard 2-dose schedule pose challenges in developing vaccination strategy (*1*). Recent studies have demonstrated effectiveness and safety of 3 doses of inactivated COVID-19 vaccine in healthy adults (*6*). In this study, we have shown a stronger sVNT response after the third dose than the second dose, as has been reported for inactivated vaccines in healthy adults, including elderly persons (*6**,**7*). Our results provide data support for the effectiveness of a 3-dose primary series of inactivated COVID-19 vaccine for all vaccinees, including PLHIV and immunocompromised hosts.

The anticipated suboptimal clinical outcome for PLHIV after COVID-19 has been shown in population-level studies (*8*) that called for prioritizing PLHIV for vaccination. With a high proportion of the global population receiving inactivated COVID-19 vaccines, we note a need to strategically adjust the regimen to attain a sustained and enhanced response in PLHIV. Routine administration of a 3-dose primary series of inactivated vaccines is a possible approach for reducing virus transmission and associated severe disease in healthy adults and PLHIV alike, as highlighted in guidance from the World Health Organization Strategic Advisory Group of Experts on Immunization (https://www.who.int/publications/i/item/WHO-2019-nCoV-vaccines-SAGE_recommendation-Sinovac-CoronaVac-2021.1) and the Centers for Disease Control and Prevention (https://www.cdc.gov/hiv/basics/covid-19.html). Recent studies have shown that the effectiveness of current COVID-19 vaccines against new variants, such as Omicron, could be reduced; immunogenicity was lower after 2 doses of inactivated vaccines than of mRNA vaccines (*9*). Further research is needed as the COVID-19 pandemic continues to evolve; in particular, the ongoing Ubuntu trial (https://www.coronaviruspreventionnetwork.org) may provide evidence for enhancing vaccination strategy for PLHIV amid the emergence of new variants.

Appendix Additional information about 3-dose primary series of inactivated COVID-19 vaccine for persons living with HIV, Hong Kong.
