# Supplementary material for: Three-Dose Primary Series of Inactivated COVID-19 Vaccine for Persons Living with HIV, Hong Kong
Source: Emerg Infect Dis. 2022 Oct;28(10):2130–2. doi: 10.3201/eid2810.220691 (PMC9514347; doi:10.3201/eid2810.220691)
Supplement: Appendix — Additional information about 3-dose primary series of inactivated COVID-19 vaccine for persons living with HIV, Hong Kong. [file 22-0691-Techapp-s1.pdf]

Article DOI: https://doi.org/10.3201/eid2810.220691

Three-Dose Primary Series of Inactivated COVID-19 Vaccine for Persons Living with HIV, Hong Kong

Appendix

Data Analysis

This is a prospective study with subject recruitment at two out of three major public HIV specialist clinics in Hong Kong. PLHIV aged 18 or above attending either clinic, receive at least two doses of CoronaVac, at least one sample collected within 90 days from either the second or third dose, and not diagnosed with COVID-19 before blood sampling were included in this study. The sVNT level within 90 days following the second dose was compared with the third dose by Mann-Whitney U test, while difference of neutralizing antibody positivity (sVNT ≥30%) in the two time points was compared in the univariable logistic regression model. The association between each independent factor and sVNT level was examined in univariable linear regression, and the factors significantly associated with sVNT level were included in the multivariable linear regression. All statistical analyses were performed in SPSS Statistics 25 (https://www.ibm.com/products/spss-statistics). Complete case analysis was performed.

Appendix Table 1. Characteristics of PLHIV receiving CoronaVac inactivated vaccine, Hong Kong

Characteristic	n (%)
Sex, n = 116	
F	16 (14)
M	100 (86)
Median age (IQR), n = 117	49 (40–56.5)
Median nadir/baseline CD4, cells/µL (IQR), n = 99	203 (54–338)
Median latest CD4, cells/µL (IQR), n = 100	564.5 (394–733)
Latest viral load status, n = 100	
≥200 copies/mL	4 (4)
<200 copies/mL	96 (96)
On antiretroviral therapy at blood sampling	
No	0 (0)
Yes	122 (100)

*Values are no. (%) except as indicated. IQR, interquartile range; PLHIV, persons living with HIV.

Appendix Table 2. Factors associated with sVNT level in study of PLHIV receiving CoronaVac inactivated vaccine, Hong Kong*

	Univariable linear regression			Multivariable linear regression		
Characteristic	B	Beta	p value	B	Beta	p value
Male sex (female coded as 0)	-3.12	-0.04	0.65	/		
Age at sampling (year-old)	0.27	0.11	0.21	/		
Third dose vaccination (second dose vaccination coded as 0)	34.11	0.58	<0.001†	33.61	0.546	<0.001†
Days from respective dose	-0.24	-0.21	0.01†	-0.17	-0.156	0.047†
Latest CD4 count (cells/μL)	0.02	0.20	0.04†	0.02	0.182	0.02†
Viral load suppression (<200/mL) (viral load ≥200/mL coded as 0)	16.89	0.12	0.22	/		
Constant (for multivariable linear regression)	/			38.47		<0.001†

*B, unstandardized coefficients; Beta, standardized coefficients.
†p<0.05
